# Unbiased post-error slowing in interference tasks: A confound and a simple solution

**DOI:** 10.3758/s13428-021-01673-8

**Published:** 2021-10-28

**Authors:** Jan Derrfuss, Claudia Danielmeier, Tilmann A. Klein, Adrian G. Fischer, Markus Ullsperger

**Affiliations:** 1 School of Psychology, University of Nottingham, University Park, Nottingham, NG7 2RD UK; 2 Institute of Psychology, Otto von Guericke University Magdeburg, Magdeburg, Germany; 3 Center for Behavioral Brain Sciences, Magdeburg, Germany; 4 Department of Neurology, Max Planck Institute for Human Cognitive and Brain Sciences, Leipzig, Germany; 5 Department of Education and Psychology, Free University, Berlin, Germany

**Keywords:** Performance monitoring, Action errors, Post-error adaptations, Cognitive control

## Abstract

We typically slow down after committing an error, an effect termed post-error slowing (PES). Traditionally, PES has been calculated by subtracting post-correct from post-error RTs. Dutilh et al. (*Journal of Mathematical Psychology, 56*(3), 208-216, 2012), however, showed PES values calculated in this way are potentially biased. Therefore, they proposed to compute robust PES scores by subtracting pre-error RTs from post-error RTs. Based on data from a large-scale study using the flanker task, we show that both traditional and robust PES estimates can be biased. The source of the bias are differential imbalances in the percentage of congruent vs. incongruent post-correct, pre-error, and post-error trials. Specifically, we found that post-correct, pre-error, and post-error trials were more likely to be congruent than incongruent, with the size of the imbalance depending on the trial type as well as the length of the response-stimulus interval (RSI). In our study, for trials preceded by a 700-ms RSI, the percentages of congruent trials were 62% for post-correct trials, 66% for pre-error trials, and 56% for post-error trials. Relative to unbiased estimates, these imbalances inflated traditional PES estimates by 37% (9 ms) and robust PES estimates by 42% (16 ms) when individual-participant means were calculated. When individual-participant medians were calculated, the biases were even more pronounced (40% and 50% inflation, respectively). To obtain unbiased PES scores for interference tasks, we propose to compute unweighted individual-participant means by initially calculating mean RTs for congruent and incongruent trials separately, before averaging congruent and incongruent mean RTs to calculate means for post-correct, pre-error and post-error trials.

## Introduction

For successful goal-directed behavior, it is important to adjust our actions when they are not going according to plan, for instance, when we have committed an error. There are several neural and behavioral post-error adjustments (Danielmeier & Ullsperger, 2011; Ullsperger et al., 2014), with post-error slowing (PES), a slowing of reaction times after errors, being the most frequently reported behavioral modulation. Interestingly, it has recently been shown that smaller PES effects in the lab are associated with more self-control failures in real life (Kronke et al., 2018). Furthermore, changes in PES have been associated with a range of different developmental or mental disorders. Altered PES effects have been shown in children with autism spectrum disorders (Sokhadze et al., 2010; Vlamings et al., 2008) and ADHD (Balogh & Czobor, 2016; Klein et al., 2013; Schachar et al., 2004), as well as in borderline personality disorders (Saunders et al., 2016), and in methamphetamine addicts (Liang et al., 2018). PES effects in individuals diagnosed with schizophrenia are mixed, with some studies showing reduced or absent PES (Alain et al., 2002; Carter et al., 2001; Kerns et al., 2005; Moran et al., 2018), while others report intact (Kopp & Rist, 1999; Laurens et al., 2003; Mathalon et al., 2002) or even increased PES (Nunez Castellar et al., 2012). Recently, post-error adjustments became part of the research domain criteria for investigating cognitive control in mental disorders (https://www.nimh.nih.gov/research-priorities/rdoc/constructs/rdoc-matrix.shtml; Kozak & Cuthbert, 2016). As PES is being used to characterize and evaluate neurodevelopmental and mental health disorders, it is important that this measure is as precise and unbiased as possible.

As noted by Schroder et al. (2020), to date there is no standard way to calculate PES. Traditionally, PES has been calculated by subtracting the mean post-correct RT (RT_post-correct_) from the mean post-error RT (RT_post-error_), with the constraint that post-correct and post-error trials must also be correct trials. However, as pointed out by Dutilh et al. (2012), PES calculated with this traditional method might be biased due to performance fluctuations: depending on how a participant's performance changes over the course of the experiment, one could either obtain spuriously increased or decreased PES values. For instance, if a participant gets tired towards the end of the experiment, more errors will occur, and RTs will be slower. Thus, most post-error trials will come from the last part of the experiment when RTs were slower overall, whereas most post-correct trials will come from earlier phases of the experiment when RTs were generally faster. With the traditional method of calculating PES, this situation would lead to inflated PES values. Another example described by Dutilh et al. (2012), that could lead to spuriously diminished PES values, would be if an individual shows changes in response caution over the course of the experiment. In phases where a participant responds more carefully, hardly any errors might occur, but response times would be quite long. If this individual later responds less carefully, RTs might get shorter, but response accuracy might drop as well. In this scenario, most post-error trials would originate from a phase with shorter RTs overall, whereas most post-correct trials would originate from phases with longer RTs, leading to decreased PES values with PES_traditional_. In line with this, a recent study by Schroder et al. (2020) concluded that PES_traditional_ tends to underestimate the magnitude of PES. Both confounds can occur when errors are not uniformly distributed across the experiment. To counteract these confounds, Dutilh et al. (2012) suggested a different quantification method, PES_robust_, where only those post-correct trials that are also pre-error trials are taken into account (PES_robust_ = RT_post-error_ - RT_pre-error_). Thus, post-error and post-correct trials will originate from the same time periods within an experiment.

While the method of calculating PES_robust_ as described by Dutilh et al. (2012) addresses potential biases caused by the fact that errors might not be spread out evenly across an experiment, it does not explicitly address a potential bias that arises when researchers use interference tasks (such as the flanker task or the Stroop task) to study performance monitoring (Dutilh et al. used a task without an interference component). Of importance for the remainder of this article, when researchers have employed interference tasks to study PES in the past, PES was not calculated separately for congruent and incongruent trials, but as composite measure across congruency conditions. Of course, reaction times in congruent trials are typically shorter than reaction times in incongruent trials. Due to this difference in RTs, a difference in the ratio of congruent and incongruent trials before and after errors will lead to biased PES estimates (see Table 1). Such an imbalance could be a consequence of the conflict adaptation effect (Gratton et al., 1992). Gratton et al. showed that the congruency of the previous trial can modulate RTs and error rates of the current trial and that error rates tend to be higher after congruent than after incongruent trials (see also Pastotter et al., 2013; Van der Borght et al., 2014).

**Table 1:** Hypothetical example illustrating the effect of an imbalance in congruent and incongruent trial numbers on robust PES estimation. In this example, the participant made 20 errors, so there are 20 pre-error and 20 post-error trials. For simplicity, we assume that all congruent RTs before errors were 1,000 ms long, that all incongruent RTs before errors were 1,200 ms long, and that the participant slowed down 100 ms in each condition after they committed an error. A) If there is no imbalance, a simple averaging approach arrives at the correct PES estimate. B) If there is an imbalance (in this example, there are relatively more congruent pre-error trials than post-error trials), a simple averaging approach overestimates the PES effect. C) This bias is absent if the RTs for the individual conditions are averaged before pre-error and post-error means are calculated

	Pre-error trials	Post-error trials	PES_robust_
A) Equal percentage of con and incon trials before and after errors	con: 10 × 1,000 ms	con: 10 × 1,100 ms	
	incon: 10 × 1,200 ms	incon: 10 × 1,300 ms	
	mean: 1,100 ms	mean: 1,200 ms	100 ms
B) Imbalance (no bias correction)	con: 15 × 1,000 ms	con: 10 × 1,100 ms	
	incon: 5 × 1,200 ms	incon: 10 × 1,300 ms	
	mean: 1,050 ms	mean: 1,200 ms	150 ms
C) Imbalance (with bias correction)	con: 15 × 1,000 ms	con: 10 × 1,100 ms	
	incon: 5 × 1,200 ms	incon: 10 × 1,300 ms	
	con mean: 1,000 ms	con mean: 1,100 ms	
	incon mean: 1,200 ms	incon mean: 1,300 ms	
	mean of means: 1,100 ms	mean of means: 1,200 ms	100 ms

Abbrev.: *con* congruent, *incon* incongruent, *PES*_*robust*_ robust post-error slowing

For traditional PES analyses, post-correct trials are used as a baseline. When post-correct RTs in interference tasks were computed in past studies, typically all post-correct trial RTs were indiscriminately averaged. However, assuming 50% of the trials are congruent and 50% are incongruent, there is some potential for bias here as well: Because error rates are higher in incongruent trials, there will be more congruent than incongruent *post-correct* trials. To illustrate this point, imagine a participant performs 101 trials and commits an error on 20 trials, 16 of which are incongruent and four congruent. Assuming trial order is random, post-error trials should on average be equally frequently congruent and incongruent. This leaves us with up to 60 post-correct trials for analysis (101 trials – (20 error trials + 20 post-error trials + first trial)). Of these, 36 will be congruent (50 congruent trials – (four congruent error trials + 10 congruent post-error trials)) and only 24 incongruent (50 incongruent trials – (16 incongruent error trials + 10 incongruent post-error trials)). Thus, simply averaging all post-correct trials will result in a biased estimate. Finally, estimates of post-error RTs might be biased as well: Again, there might be more congruent than incongruent post-error trials, due to the fact that an incongruent post-error trial is more likely to be incorrect and would thus be excluded from the trials eligible as post-error trials.

In the present article, using data from a large-scale study of the Eriksen flanker task (Danielmeier et al., 2009; Eriksen & Eriksen, 1974), we set out to investigate biases in traditional and robust PES when pre-error, post-correct, and post-error RTs are calculated without taking imbalances in the percentage of congruent and incongruent trials into account. We will investigate if these imbalances depend on response-stimulus intervals (RSIs). Taking RSI into account for PES calculations is important as it has previously been shown that PES strongly depends on RSIs, with shorter RSIs leading to more PES (Buzzell et al., 2017; Danielmeier & Ullsperger, 2011; Jentzsch & Dudschig, 2009). Therefore, our analysis will match the RSI length before post-error trials and the RSI before post-correct trials (traditional PES)/pre-error trials (robust PES) when calculating PES. In addition, we will investigate the degree of bias in PES for when means or medians are calculated at the level of individual participants. Finally, we will use synthetic data to explore biases across a range of imbalances and interference effects. Throughout the paper, we will show that these biases can easily be corrected by averaging congruent and incongruent trials separately before averaging these condition-specific means to calculate an overall mean.

## Method

### Participants

This study is based on a dataset that has been reported elsewhere (Fischer et al., 2016; Fischer et al., 2018). The initial sample consisted of 803 individuals (409 males, 394 females; age range: 18–40 years, mean age: 23.99 years; SD: 3.89; 33 left-handers). A total of 388 datasets were collected at the Radboud University of Nijmegen, all remaining datasets were collected at the Max Planck Institute for Human Cognitive and Brain Sciences in Leipzig, using identical code and equipment. Study procedures were approved by the ethics committees in Nijmegen (ECG04032011) and at the University of Leipzig (285-09-141209). To be included in the study, participants needed to meet the following criteria: no history of psychiatric or neurological disease; no regular use of psychotropic medication; no relevant history of drug abuse (consumption within the last month or more than five times in lifetime; without cannabis); no regular consumption of cannabis (more than three times per month); no alcohol intake on the day of the study. Our task performance criteria (see below for details) led to the exclusion of 450 individuals for the analysis based on individual-participant means. Thus, the final sample consisted of 353 individuals (172 males, 181 females) between 18 and 37 years of age (mean: 23.59 years, SD: 3.66), 13 of whom were left-handed. For the analysis based on individual-participant medians, 385 participants were excluded, resulting in a final sample size of 418 individuals (197 males, 221 females; mean age: 23.67 years, SD: 3.72, range: 18 to 38 years, 18 left-handed).

### Task

A speeded arrow-version of the Eriksen Flanker task was employed (for a figure illustrating the task, see Fischer et al., 2018). Participants were instructed to respond as quickly and accurately as possible to the direction (left or right) of a centrally presented target arrow that appeared on screen for 33 ms. They responded with their left or right thumb, corresponding to the arrow direction, on a custom-made hand-held response box. Participants were also instructed to ignore four flanking arrows that appeared above and below the target 83 ms earlier. The size of all arrows was 1.9° × 1.3° of visual angle. The task consisted of 1088 trials, with a self-paced break after every 200 trials. On half of the trials, the direction of flankers and target was the same (congruent trials), whereas they pointed in opposite directions on the other half (incongruent trials). The distance between flanker and target stimuli was either close (flanker-target distance: 3.5° and 1.75° for distal and proximal distractor arrows, respectively) or far (6.5° and 4°). The time between the response and the onset of the next trial (response-stimulus interval, RSI) was either short (250 ms) or long (700 ms). Half of the congruent trials were preceded by a short and the other half by a long RSI (same for incongruent trials), and half of the far trials were preceded by a short, and the other half by a long RSI (same for close trials). Thus, the task combined the factors flanker-target congruency (congruent or incongruent), RSI (short or long) and flanker-target distance (close or far). The trial order was pseudo-random with counterbalanced transition probabilities for congruency and flanker-target distance.

### Procedure

The flanker task data were acquired within a larger project in the context of a set of other tasks (time estimation task, oddball task, resting state EEG measure) that are not reported here. Electroencephalography data were collected simultaneously but are not included here. The order of the different tasks was randomized between participants. Participants sat in a dimly lit EEG recording chamber while performing the task. The overall task duration across all different tasks was roughly 90 min. Written informed consent was obtained from each participant before inclusion in the study and subjects received either financial compensation or course credits.

### Data analysis

The analyses reported here focus exclusively on close trials as studies employing the flanker task typically present flankers directly adjacent to the target. Our data and analysis repository (see below) also includes an analysis of the far trials.

Trial types were defined as follows: *Error trials* were trials with incorrect responses (i.e., trials with no response before the deadline were not taken into account for the present analyses). *Post-correct trials* were correct trials that followed another correct trial. *Pre-error trials* were correct trials that followed a correct trial and preceded an error. *Post-error trials* were correct trials that followed an error and preceded a correct trial. These definitions made sure that single correct trials between two errors were excluded from the analysis (categorization for these trials would be ambiguous as they were both post-error and pre-error trials).

We calculated four types of PES: uncorrected traditional PES, corrected traditional PES, uncorrected robust PES, and corrected robust PES. For each participant, trials relevant for the analyses were categorized along three dimensions: trial type (post-correct, pre-error, post-error; note that some post-correct trials are also pre-error trials), congruency (congruent, incongruent), and RSI (short, long). The relevant RSI was always the RSI that *preceded* a given trial. For example, when referring to a pre-error trial as a "short RSI" trial, this indicates that the pre-error trial was preceded by a short RSI.

We performed two different sets of analyses, one based on mean RTs and one based on median RTs on the individual-subject level. For the mean-based analysis, the different types of PES were calculated for a given participant as follows:

1 $$ {\displaystyle \begin{array}{l}{\mathrm{PES}}_{trad, uncorr}={\overline{\mathrm{RT}}}_{post- error}-{\overline{\mathrm{RT}}}_{post- correct}\\ {}{\mathrm{PES}}_{trad, corr}=\left(\frac{{\overline{\mathrm{RT}}}_{post- error, incon}+{\overline{\mathrm{RT}}}_{post- error, con}}{2}\right)-\left(\frac{{\overline{\mathrm{RT}}}_{post- correct, incon}+{\overline{\mathrm{RT}}}_{post- correct, con}}{2}\right)\\ {}\begin{array}{l}{\mathrm{PES}}_{robust, uncorr}={\overline{\mathrm{RT}}}_{post- error}-{\overline{\mathrm{RT}}}_{pre- error}\\ {}{\mathrm{PES}}_{robust, corr}=\left(\frac{{\overline{\mathrm{RT}}}_{post- error, incon}+{\overline{\mathrm{RT}}}_{post- error, con}}{2}\right)-\left(\frac{{\overline{\mathrm{RT}}}_{pre- error, incon}+{\overline{\mathrm{RT}}}_{pre- error, con}}{2}\right)\end{array}\end{array}} $$

For uncorrected PES scores, congruent and incongruent trials were first concatenated and then averaged. For corrected PES scores, congruent and incongruent RTs were initially averaged separately, thus removing any bias caused by imbalances in trial numbers. To calculate PES for the group, individual participant PES scores were averaged. All calculations were done for each RSI separately.

For the median-based analysis, medians were calculated on the individual-participant level. For uncorrected PES scores, congruent and incongruent trials were concatenated before the median was calculated. For corrected PES scores, medians for congruent and incongruent RTs were calculated separately before averaging the two medians. To calculate PES for the group, individual participant PES scores were averaged. All calculations were done for each RSI separately.

In our analyses, we removed trials immediately following breaks, and all trials with RTs below 100 ms or above 1500 ms. To be included in the analysis, a participant was required to have at least five congruent and incongruent pre-error and post-error trials in each RSI condition (short and long; i.e., a minimum of 20 errors in close trials, corresponding to a minimum error rate of 3.7%). For the analysis based on individual participant means, we removed outlier RTs using the median absolute deviation (scale factor 1.4826, threshold 2.5; Leys et al., 2013). As the median is insensitive to outliers, outlier rejection was not performed for the analysis based on individual participant medians. Overall, 425 participants in the mean-based analysis and 504 participants in the median-based analysis had sufficient trial numbers (as no outliers RTs were rejected, more participants achieved the five-trial criterion in the median-based analysis). We also investigated if there were any participants who did not show an overall interference effect in correct trials, or made more errors in congruent as compared to incongruent trials. No such participants were identified. As a final criterion, we required a minimum accuracy of 80% on congruent trials, and 60% on incongruent trials. Based on this criterion, for the mean-based analysis a further 72 participants were excluded, resulting in the final sample size of 353 participants. For the median-based analysis, a further 86 participants were excluded, resulting in the final sample size of 418 participants.

Data were analyzed using custom-written scripts in Python (v. 3.7.7) using Jupyter notebook (v. 6.0.3, https://jupyter.org/), Pandas (v. 1.0.3, https://pandas.pydata.org/), seaborn (v. 0.10.1, https://seaborn.pydata.org/), and pingouin (v. 0.3.11, https://pingouin-stats.org/). Error bars shown in figures were calculated using seaborn and correspond to 95% multilevel bootstrapped CIs based on 1000 iterations. The multilevel bootstrapping approach first resampled participants and then observations within participants to account for the repeated-measures design. The kernel density estimates were calculated using the default settings in seaborn. The data and analysis scripts are available at 10.24352/ub.ovgu-2021-024.

## Results

### Pre-error trials

**Fig. 1:**
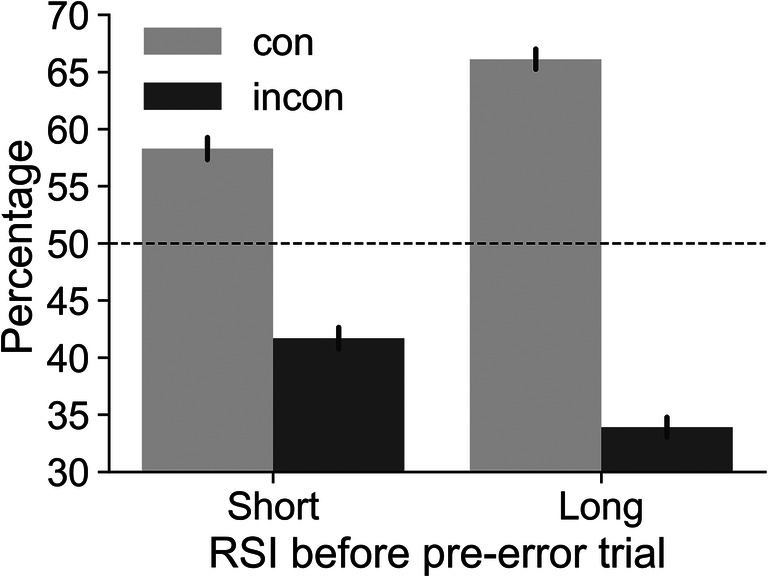
Percentage of congruent (con) and incongruent (incon) pre-error trials depending on the response-stimulus interval (RSI) before the pre-error trial. The dashed line at 50% represents the expected percentages when no imbalance is present. Results show that pre-error trials were more likely to be congruent, especially when the RSI before the pre-error trial was long. Error bars represent 95% CIs

Robust PES uses pre-error trials as a baseline. Therefore, we initially investigated if there was an imbalance in the number of congruent and incongruent pre-error trials. We found that this was indeed the case (see Fig. 1). Pre-error trials were more frequently congruent, both when the RSI before the pre-error trial was short (58.3% congruent), and when it was long RSI (66.1% congruent). A repeated-measures ANOVA showed that this imbalance was significant: There was a main effect of congruency, *F*(1, 352) = 1077, *p* < .001, ${\eta}_p^2$ = 0.75, and an interaction of congruency x RSI, *F*(1, 352) = 175, *p* < .001, ${\eta}_p^2$ = 0.33 (as the average percentage for each RSI must be 50%, there can be no main effect of RSI). Post hoc *t* tests showed that the percentage of congruent trials as compared to incongruent trials was higher at both RSIs. In addition, the percentage of congruent trials was higher at the long RSI as compared to the short RSI (all post hoc *p*'s < .001). These results indicate that an error is more likely preceded by a congruent trial than an incongruent trial, in particular if the RSI before the pre-error trial is long.

Note that these results refer to the RSI *before the pre-error trial*, not the RSI before the error trial. We focus on the RSI before the pre-error trial because it is this RSI that needs to be matched to the RSI before the post-error trial for robust PES calculations. For example, RTs in post-error trials preceded by short RSIs should be compared to RTs in pre-error trials also preceded by short RSIs. Below, we show in more detail that RTs in general as well as PES in particular depend on RSI (see Table 3).

Which factors are driving the imbalance in favor of congruent pre-error trials? To briefly reiterate, when calculating PES a pre-error trial is a correct trial that follows another correct trial (otherwise the categorization would be ambiguous as the trial in question would be both a pre-error and a post-error trial) and precedes an error trial. We can investigate the impact of these constraints on the imbalance by successively removing them. This is shown in Table 2. The results show that removing constraints reduces the imbalance in favor of congruent trials at short and long RSIs. However, even when neither the pre-error nor the error–2 trial must be correct, pre-error trials are still more likely to be congruent. In our study, the error rate after long RSI congruent trials was 18.6 vs. 14.9% after long RSI incongruent trials, in line with the conflict adaptation effect.

**Table 2.** Percentages of congruent pre-error trials for the post-error slowing analysis and when relaxing constraints required for the post-error slowing analysis

Constraints	Percentage of congruent pre-error trials	
	Short RSI	Long RSI
Pre-error trial and error–2 trial correct (see Fig. 1)	58.3	66.1
Pre-error trial correct (but not error–2 trial)	58.2	65.7
Error–2 trial correct (but not pre-error trial)	54.6	57.8
Neither pre-error nor error–2 trial must be correct	54.5	57.8

### Post-correct trials

**Fig. 2:**
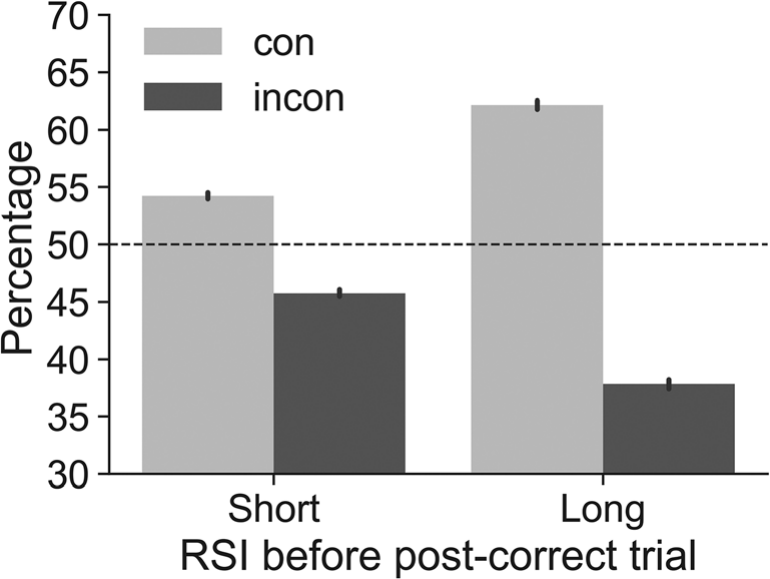
Percentage of congruent (con) and incongruent (incon) post-correct trials depending on the response-stimulus interval (RSI) before the post-correct trial. The dashed line at 50% represents the expected percentages when no imbalance is present. Results show that post-correct trials were more likely to be congruent, especially when the RSI before the pre-error trial was long. Error bars represent 95% CIs

Traditional PES uses post-correct trials which are themselves correct as a baseline. As error rates for incongruent trials are higher (29.9 vs. 5.0% for congruent), there will also be more congruent than incongruent correct post-correct trials (see Introduction). Figure 2 depicts the imbalance for post-correct trials preceded by short or long RSIs. As expected, post-correct trials were more frequently congruent, both when the RSI before the pre-error trial was short (54.2% congruent), and when it was long RSI (62.2% congruent). A repeated-measures ANOVA showed that this imbalance was significant: There was a main effect of congruency, *F*(1, 352) = 3653, *p* < .001, ${\eta}_p^2$ = 0.91, and an interaction of congruency x RSI, *F*(1, 352) = 1299, *p* < .001, ${\eta}_p^2$ = 0.79. Post hoc *t* tests showed that the percentage of congruent trials as compared to incongruent trials was higher at both RSIs. In addition, the percentage of congruent trials was higher at the long RSI as compared to the short RSI (all post hoc *p*'s < .001).

### Post-error trials

**Fig. 3:**
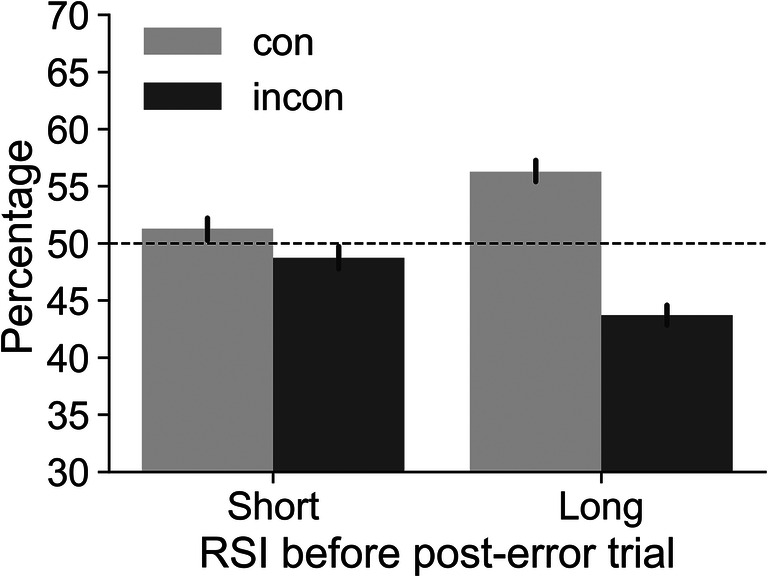
Percentage of congruent (con) and incongruent (incon) post-error trials depending on the response-stimulus interval (RSI) before the post-error trial. The dashed line at 50% represents the expected percentages when no imbalance is present. Results show that post-error trials tended to be congruent, especially when the RSI before the post-error trial was long. Error bars represent 95% CIs

Next, we investigated if there was also an imbalance for post-error trials. We found that also post-error trials tended to be congruent. For short RSIs, 51.3% of the trials were congruent, and for long RSIs, 56.3% of the trials were congruent (Fig. 3). A repeated-measures ANOVA showed that this imbalance was significant: There was a main effect of congruency, *F*(1, 352) = 117, *p* < .001, ${\eta}_p^2$ = 0.25, and an interaction of congruency x RSI, *F*(1, 352) = 54, *p* < .001, ${\eta}_p^2$ = 0.13. Post hoc *t* tests showed that the percentage of congruent trials as compared to incongruent trials was higher at both RSIs (although the effect at the short RSI was only just significant after Bonferroni correction at *p* = .047). In addition, the percentage of congruent trials was higher at the long RSI as compared to the short RSI (all other post hoc *p*'s < .001). Note that congruent and incongruent trials (irrespective of accuracy) occurred equally frequently after an error (e.g., 50.3% of post-error trials preceded by a long RSI were congruent). The bias is caused by the fact that PES calculations only take into account *correct* post-error trials, and correct post-error trials are more often congruent than incongruent.

Taken together, these results show that at least in a Eriksen flanker paradigm consisting of 50% congruent and 50% incongruent trials in pseudorandomized order where transition frequencies are counterbalanced, the number of congruent and incongruent pre-error trials, post-correct trials as well as post-error trials is not balanced. Thus, mean RTs that do not take this imbalance into account will be biased. In addition, as the degree of imbalance differs for pre-error/post-correct (the baseline conditions for robust and traditional PES, respectively) and post-error trials, measures computed based on these mean RTs (such as PES) will also be biased. Below we explore the consequences of not correcting this bias.

### PES based on individual-subject means

We now address the question how the imbalances described above affect PES values. For the analyses reported in this section, mean RTs (as opposed to the medians that will be considered below) were computed for individual participants. These means were then averaged to calculate group means (Table 3). As described in the *Data analysis* section, we compared four types of PES calculations: uncorrected traditional PES, corrected traditional PES, uncorrected robust PES and corrected robust PES. We found, as did Dutilh et al. (2012), that PES estimates were generally smaller when using the traditional approach (Table 3). More importantly, and in line with the imbalances reported above, our results showed that uncorrected PES estimates (i.e., PES estimates based on simple concatenation of congruent and incongruent trials) tended to be higher than corrected robust PES estimates. Comparing traditional uncorrected and corrected PES, there was no bias at the short RSI, which is in line with the fact that congruency imbalances for short RSI post-error trials and post-correct trials were small (51.3 and 54.2% congruent, respectively). However, traditional PES did overestimate PES at the long RSI (9 ms bias, 37% increase relative to the corrected value, Cohen's *d*_*Z*_ = 0.51). Comparing robust uncorrected and corrected PES, the overestimation was very small at the short RSI (4ms bias, 4.4% increase relative to the corrected value, Cohen's *d*_*Z*_ = 0.18), but more pronounced at the long RSI (16ms bias, 42% increase relative to the corrected value, Cohen's *d*_*Z*_ = 0.71).

**Table 3:** Means of mean RTs in ms (± SD) for the conditions used to calculate post-error slowing (PES)

	PES short RSI				PES long RSI			
	Trad_uncorr_	Trad_corr_	Robust_uncorr_	Robust_corr_	Trad_uncorr_	Trad_corr_	Robust_uncorr_	Robust_corr_
Post-error	459 (65)	459 (65)	459 (65)	459 (65)	374 (42)	377 (41)	374 (42)	377 (41)
Post-correct/pre-error^1^	385 (35)	385 (34)	364 (35)	368 (33)	341 (27)	353 (26)	320 (31)	339 (31)
PES	74 (48)	74 (49)	95 (52)	91 (53)	33 (31)	24 (29)	54 (36)	38 (34)

^1^Post-correct trials are used for the traditional PES calculation, pre-error trials for the robust approach.

Abbr.: RSI = response-stimulus interval; Trad = traditional PES calculation; corr = corrected; uncorr = uncorrected

**Fig. 4:**
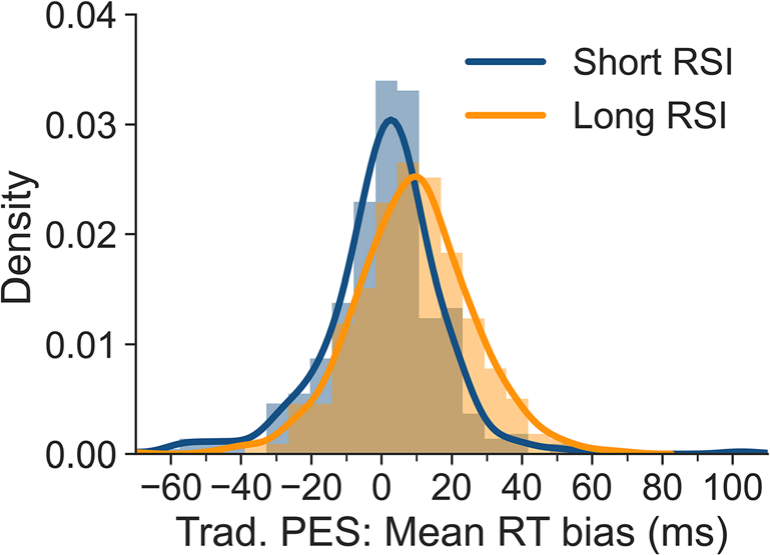
Bias for uncorrected traditional PES values for all participants based on individual-subject means, separately for short and long RSI. A bias of 0 indicates that uncorrected and corrected traditional PES values did not differ. Negative values indicate that the uncorrected approach underestimated PES, and positive values that it overestimated PES

Overall, bias for uncorrected vs. corrected traditional PES was either absent (short RSI) or moderate (long RSI). However, a moderate overall bias could be the result of more substantial misestimates on the individual participant level if the misestimates sometimes underestimate and sometimes overestimate the actual PES effect. This would be particularly problematic for studies planning correlational across-participant analyses that include PES effects as a variable as here individual PES values determine the size of the correlation. To determine individual biases, we subtracted individual PES_*trad,corr*_ from PES_*trad,uncorr*_ values. Figure 4 shows the resulting traditional PES bias for all 353 participants, separately for the short and long RSI. The means of these distributions correspond to the biases mentioned above (i.e., 0 ms and 9 ms). The SD is 18 ms for the short RSI and 17 ms for the long RSI. The results indicate that the degree of bias is somewhat variable between participants and that substantial individual misestimates occur even for short RSIs without a mean overall bias.

**Fig. 5:**
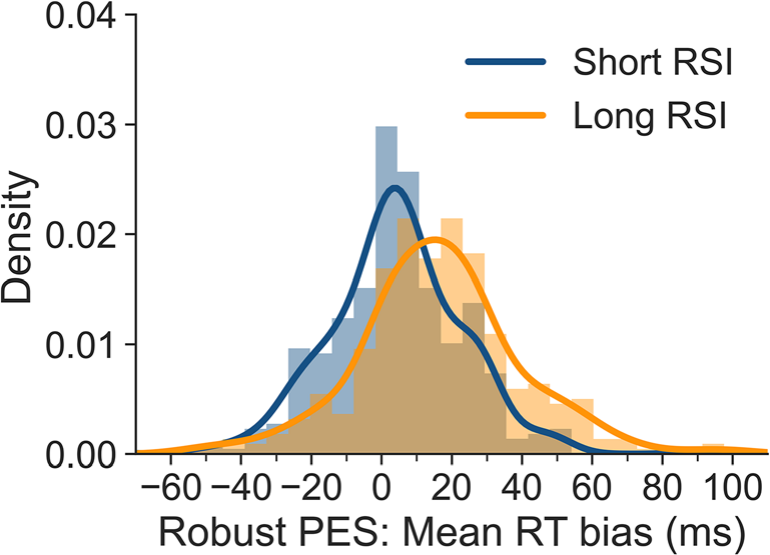
Bias for uncorrected robust PES values for all participants based on individual-subject means, separately for short and long RSI. A bias of 0 indicates that uncorrected and corrected robust PES values did not differ. Negative values indicate that the uncorrected approach underestimated PES, and positive values that it overestimated PES

To determine individual biases for robust PES, we subtracted individual PES_*robust,corr*_ from PES_*robust,uncorr*_ values. Figure 5 shows the resulting robust PES bias for all participants, separately for the short and long RSI. The means of these distributions correspond to the biases mentioned above (i.e., 4 and 16 ms). The SD is 20 ms for the short RSI and 23 ms for the long RSI. The results show that the degree of bias is highly variable between participants and that even for the short RSI substantial misestimates occur.

Taken together, these results show that uncorrected PES values tend to overestimate PES in our task. This overestimation was negligible for the short RSI, but more pronounced for the long RSI. In addition, the interindividual variability of bias is substantial, even for trials preceded by short RSIs.

### PES based on individual-subject medians

We also investigated how the calculations of medians at the individual participant level would affect the PES bias. These medians were then averaged to calculate group means (Table 4). As mentioned above, the absence of outlier RT rejection led to the inclusion of more participants in this analysis. However, this had only a very minor effect on the pre-error and post-error imbalances reported in Figs. 1, 2, and 3 (all changes < 1%) and the imbalances are therefore not displayed again. As above, we compared four types of PES calculations: uncorrected traditional PES, corrected traditional PES, uncorrected robust PES and corrected robust PES. Comparing traditional uncorrected and corrected PES, there was a small bias at the short RSI. In this case, the uncorrected measure underestimated PES (– 5 ms bias, 6.7% decrease relative to corrected value, Cohen's *d*_*Z*_ = – 0.25). However, traditional PES did overestimate PES at the long RSI (10ms bias, 40% increase relative to the corrected value, Cohen's *d*_*Z*_ = 0.40). Comparing robust uncorrected and corrected PES, there was a negligible bias of 1 ms at the short RSI. The bias at the long RSI (20 ms, 54% increase relative to corrected value, Cohen's *d*_*Z*_ = 0.67), however, was even more pronounced than for the mean-based analysis.

**Table 4:** Means of median RTs in ms (± SD) for the conditions used to calculate post-error slowing (PES)

	PES short RSI				PES long RSI			
	Trad_uncorr_	Trad_corr_	Robust_uncorr_	Robust_corr_	Trad_uncorr_	Trad_corr_	Robust_uncorr_	Robust_corr_
Post-error	458 (63)	459 (64)	458 (63)	459 (64)	374 (42)	377 (38)	374 (42)	377 (38)
Post-correct/pre-error^1^	389 (35)	385 (35)	367 (38)	369 (32)	339 (31)	352 (25)	317 (35)	340 (29)
PES	69 (47)	74 (48)	91 (51)	90 (52)	35 (34)	25 (27)	57 (41)	37 (32)

^1^ Post-correct trials are used for the traditional PES calculation, pre-error trials for the robust approach.

Abbr.: RSI = response-stimulus interval; Trad = traditional PES calculation; corr = corrected; uncorr = uncorrected

**Fig. 6:**
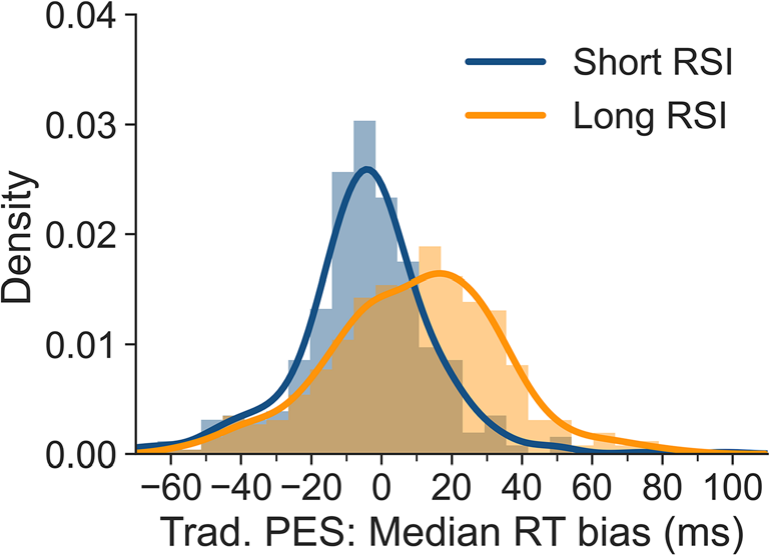
Bias for uncorrected traditional PES values for all participants based on individual-subject medians, separately for short and long RSI. A bias of 0 indicates that uncorrected and corrected traditional PES values did not differ. Negative values indicate that the uncorrected approach underestimated PES, and positive values that it overestimated PES

Again, we investigated the interindividual variability of bias. Figure 6 shows the resulting traditional PES bias when medians are calculated for individual participants, separately for the short and long RSI. The means of these distributions correspond to the biases mentioned above (i.e., – 5 ms and 10 ms). The SD is 21 ms for the short RSI and 24 ms for the long RSI. The results indicate that the degree of bias is somewhat variable between participants and that substantial individual misestimates occur even for the short RSI that has a very small overall bias.

**Fig. 7:**
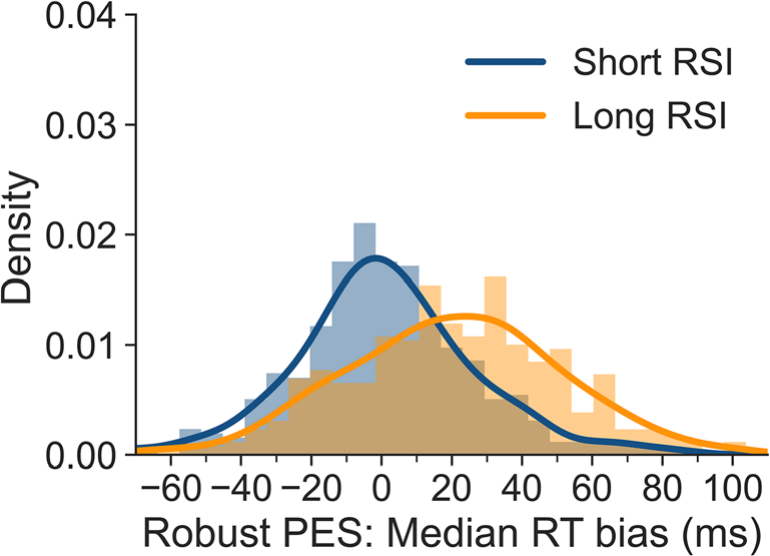
Degree of bias for uncorrected robust PES values for all participants based on individual-subject medians, separately for short and long RSI. A value of 0 indicates that uncorrected and corrected robust PES values did not differ. Negative values indicate that the uncorrected approach underestimated PES, and positive values that it overestimated PES

Figure 7 shows the uncorrected robust PES bias, separately for the short and long RSI. The means of these distributions correspond to the biases mentioned above (i.e., 1 and 20 ms). The SD is 26 ms for short RSI, and 30 ms for the long RSI. Again, these results show that the bias for individual participants is highly variable (even if, as for the short RSI, there is only negligible overall bias).

Taken together, when calculating medians on an individual-subject level, these results show that not taking the imbalance of congruent and incongruent pre-error and post-error trials into account can lead to large PES biases in the flanker task. However, even if the overall mean uncorrected robust PES effect is essentially unbiased (such as at the short RSI), individual PES estimates can nevertheless be severely biased.

### Causes of bias

The bias is a consequence of not taking the differential imbalances in congruent and incongruent trial numbers before and after errors into account, leading to the calculation of a weighted average of the two distributions. Below, we demonstrate the degree of bias for a range of different interference effects and imbalances. For these demonstrations, we make the simplifying assumptions that RTs are normally distributed and that congruent and incongruent RTs have the same SD. When the mean for an individual participant is computed without correcting for an imbalance, the degree of RT bias is a linear function of the interference effect (i.e., the RT difference for incongruent and congruent trials) and the imbalance. The bias for a given participant can be calculated using the following formula:

2 $$ {\mathrm{RT}}_{bias}=\frac{{\overline{\mathrm{RT}}}_{incon}-{\overline{\mathrm{RT}}}_{con}}{100}\times \mathrm{deviation}\ \mathrm{from}\ 50\% $$

**Fig. 8:**
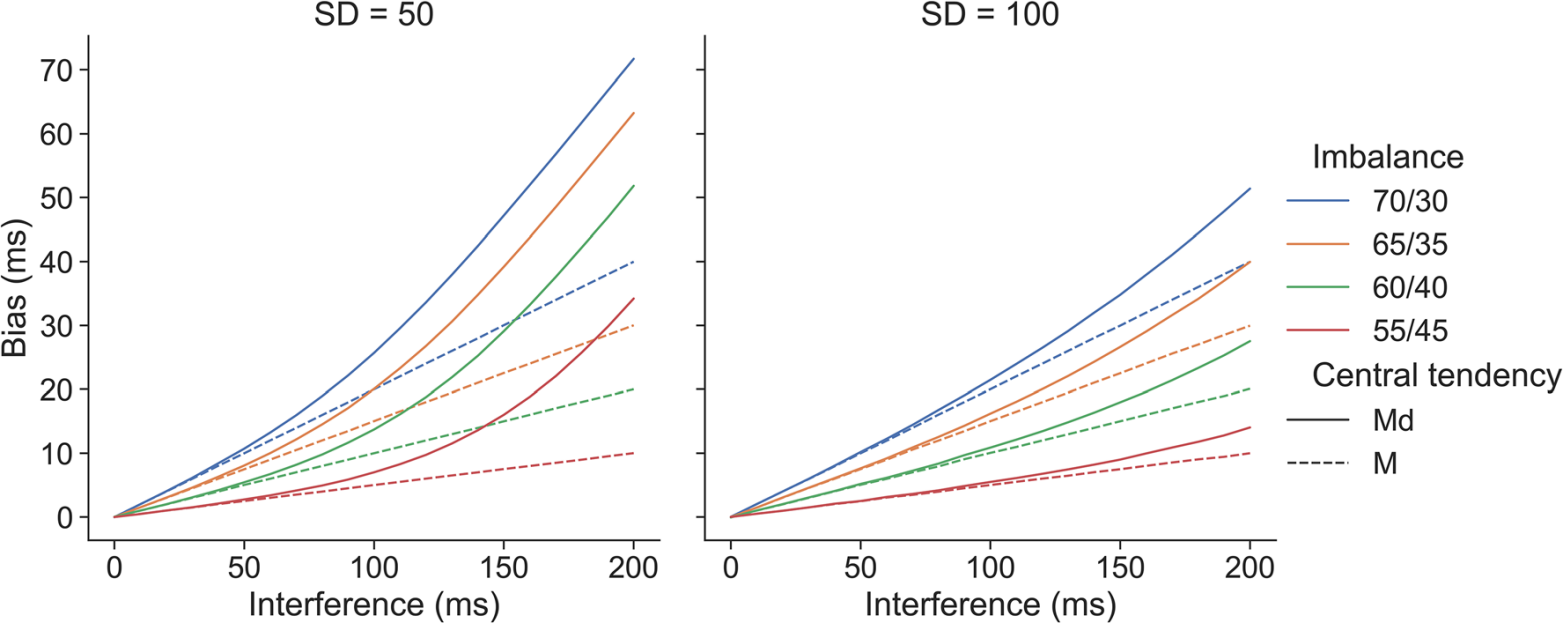
Degree of bias for mixtures of two normal distributions with different means (mean difference = Interference), imbalances (line colors), central tendencies (line types), and SDs (panels). If the central tendency measure is the mean, bias is a linear function of interference and imbalance. If the median is calculated, bias is a non-linear function of interference, imbalance, and SD. As explained in the text, all biases illustrated here can easily be corrected by calculating unweighted means

For example, an interference effect of 100 ms and an imbalance of 70% congruent and 30% incongruent trials (corresponding to a 20% deviation from 50%) will result in an RT bias of 20 ms. Figure 8 demonstrates the degree of bias for different interference effects (0–200 ms), SDs (50 and 100 ms), and imbalances (55%/45% to 70%/30% congruent/incongruent, in 5% steps). Apart from the interference effect, the range of values was chosen based on the values typically observed in our data. We extended the range of interference effects to 200 ms to show the potential bias for tasks with larger interference effects than those found in the flanker task. Note that for the mean, the bias is independent of the SD.

Our data from the flanker task showed that the PES bias was more pronounced when medians were computed as compared to means. The reason for this becomes clear when inspecting Fig. 8: In the presence of an imbalance, calculating medians always results in a degree of bias at least as strong as that observed for the mean. In addition, this bias rapidly and non-linearly increases for larger interference effects. Finally, for medians, the bias also depends on the SD, with smaller SDs leading to increased degrees of bias. That smaller SDs lead to more bias can be understood by returning to our hypothetical example from the introduction. In Table 1B, in the absence of variability, even an imbalance of a single trial would result in a median identical to the median of the distribution with more trials when the two distributions are mixed. In our example, this would lead to a median of 1000 ms for pre-error trials, thus overestimating PES by 200 ms (as compared to 150 ms when the mean is calculated). This effect becomes less pronounced if the two distributions overlap more.

## Discussion

The present article presents an approach to calculating unbiased post-error slowing (PES) for tasks in which the frequency of conditions used for calculating PES is not balanced before or after errors. Using data from a large-scale flanker study, we found that pre-error, post-correct and post-error trials were more frequently congruent than incongruent, in particular for trials preceded by long response-stimulus intervals (RSIs). For pre-error trials, this is in line with studies investigating the conflict adaptation effect. It has been shown previously that error rates are higher after congruent than after incongruent trials (e.g., Bugg, 2008; Eichele et al., 2010) meaning that more errors are preceded by congruent than incongruent trials. As reaction times typically differ between congruent and incongruent trials, these imbalances in trial types can lead to biases in PES estimation. In our paradigm, these biases were negligible for the short RSI (250 ms). However, they were more pronounced at the long RSI (700 ms). Both traditional PES estimates and robust PES estimates were affected by these biases. The uncorrected traditional approach overestimated PES at the long RSI by 37% (9 ms) when means were computed at the individual-participant level and by 40% (10 ms) when medians were calculated. The uncorrected robust approach overestimated PES at the long RSI by 42% (16 ms) when means were computed at the individual-participant level and by 54% (20 ms) when medians were calculated. We also showed that there is substantial variability in bias observed across participants (cf. Figs. 4, 5, 6 and 7), even when the overall bias is small or absent. This artefactual variability would add noise to correlational analyses, which would be a particular problem in studies with small samples. Removing this artefactual variability is in particular warranted if post-error adjustments are to be used as part of the research domain criteria for investigating cognitive control in mental disorders (Kozak & Cuthbert, 2016).

While the observed PES biases were relatively small in absolute numbers, these biases could be further amplified in paradigms that show larger interference effects than the flanker task or in paradigms that use longer RSIs (cf. Fig. 8). At present, we can only speculate about how pronounced the biases might be for other interference paradigms such as the Stroop task. However, all interference paradigms combine easier and harder conditions, thus it can be assumed that other paradigms will also present with an overrepresentation of correct trials coming from the easier condition. Moreover, the conflict adaptation effect is assumed to be a general feature of our cognitive system (e.g., Botvinick et al., 2001; Ullsperger et al., 2014), which would again suggest that similar imbalances will be found with other paradigms.

Importantly, it is not possible to avoid the observed PES biases by careful counterbalancing of conditions before the experiment. The biases are a consequence of differences in speed and accuracy associated with the experimental conditions. Thus, the confounds result from the behaviour of the participants, not from confounds in ordering conditions.

Fortunately, correcting for the confounds by calculating unbiased PES estimates is straightforward. At the heart of the bias lies the calculation of PES based on mixtures of unequally weighted distributions (i.e., a larger number of fast congruent trials is combined with a smaller number of slow incongruent trials before the mean is computed). To remove the bias, means should be computed separately for congruent and incongruent trials, before averaging these means to calculate an overall mean (see Steinhauser & Andersen, 2019, for a related approach).

As mentioned previously, the bias we describe is only an issue for paradigms with multiple conditions. If all pre-error and post-error trials belong to the same condition (see Dutilh et al., 2012), there can be no imbalance. Notably, using paradigms with multiple conditions has another consequence: It is possible that pre-error and post-error trials belong to different conditions. For example, in our study the pre-error trial could have been preceded by a long RSI and the post-error trial by a short RSI. This issue is not specific to our unbiased robust PES approach, but applies to any approach using pre-error and post-error trials to calculate PES in a paradigm with more than one condition. Could this create confounds similar to the ones described by Dutilh et al. (2012) for traditional PES calculations? Specifically, Dutilh et al. argued that decreases in motivation over time could lead to spurious PES and decreases in response caution over time to spurious post-error speeding. We argue that confounds analogous to those for traditional PES calculations do not exist for robust PES calculations in paradigms with multiple conditions. For traditional PES calculations, the confounds exist because the frequency with which post-correct trials and post-error trials occur can vary across the course of the experiment. For example, in the case of spurious PES, many post-error RTs will come from the later phase of the experiment where the participant was less motivated and produced slow responses and many errors. This is different for the robust PES approach for paradigms with multiple conditions: Even though the pre-error and post-error trials might not belong to the same condition, both will always be directly adjacent to an error and thus cannot be derived from different phases of the experiment where motivation or response caution differed.

In conclusion, our results show that traditional and robust measures of PES in flanker tasks are likely biased if congruent and incongruent trials are combined and averaged. This bias stems from the fact that (a) congruent trials are faster than incongruent trials and (b) the percentage of congruent trials differs across conditions used to calculate PES (i.e., post-correct, pre-error and post-error trials). A simple solution to correcting this confound is to separately average congruent and incongruent RTs before calculating an overall average.

## Data Availability

The data are available at 10.24352/ub.ovgu-2021-024
